# Evaluating the Cost-Effectiveness of Lifestyle Modification versus Metformin Therapy for the Prevention of Diabetes in Singapore

**DOI:** 10.1371/journal.pone.0107225

**Published:** 2014-09-09

**Authors:** May Ee Png, Joanne Su-Yin Yoong

**Affiliations:** National University of Singapore, Saw Swee Hock School of Public Health, Singapore, Singapore; Sezione di Gastroenterologia, Italy

## Abstract

**Background:**

In Singapore, as diabetes is an increasingly important public health issue, the cost-effectiveness of pursuing lifestyle modification programs and/or alternative prevention strategies is of critical importance for policymakers. While the US Diabetes Prevention Program (DPP) compared weight loss through lifestyle modification with oral treatment of diabetes drug metformin to prevent/delay the onset of type 2 diabetes in pre-diabetic subjects, no data on either the actual or potential cost effectiveness of such a program is available for East or South-east Asian populations. This study estimates the 3-year cost-effectiveness of lifestyle modification and metformin among pre-diabetic subjects from a Singapore health system and societal perspective.

**Methodology:**

Cost effectiveness was analysed from 2010–2012 using a decision-based model to estimate the rates of getting diabetes, healthcare costs and health-related quality of life. Cost per quality-adjusted life year (QALY) was estimated using costs relevant to the time horizon of the study from Singapore. All costs are expressed in 2012 US dollars.

**Principal Findings:**

The total economic cost for non-diabetic subjects from the societal perspective was US$25,867, US$28,108 and US$26,177 for placebo, lifestyle modification and metformin intervention respectively. For diabetic patients, the total economic cost from the societal perspective was US$32,921, US$35,163 and US$33,232 for placebo, lifestyle modification and metformin intervention respectively. Lifestyle modification relative to placebo is likely to be associated with an incremental cost per QALY gained at US$36,663 while that of metformin intervention is likely to be US$6,367 from a societal perspective.

**Conclusion:**

Based on adaptation of the DPP data to local conditions, both lifestyle modification and metformin intervention are likely to be cost-effective and worth implementing in Singapore to prevent or delay the onset of type 2 diabetes. However, the cost of lifestyle modification from the societal perspective would have to be reduced in order to match the cost-effectiveness of metformin intervention.

## Introduction

Diabetes is a chronic metabolic disease defined by high blood glucose levels with costly long-term complications. The global prevalence of diabetes was 8.3% in 2011 is and expected to increase to 9.9% in 2030 among adults aged 20–79 years old [1]. This increase in prevalence, coupled with inflation, has contributed significantly to the increase in economic costs attributable to diabetes [2]. In Singapore, diabetes is one of the top ten causes of death and its prevalence among adults is projected to increase from 11.3% in 2010 to 15% in 2050 [3]–[5].

Lifestyle modification has been advocated to reduce risk of developing diabetes as patients often have lifestyle habits that lead to their problem [6]. However, drug therapy may be an important alternative strategy for preventing type 2 diabetes when lifestyle modifications fail or are infeasible. This is demonstrated by the Diabetes Prevention Program (DPP) which looked at both lifestyle modification and metformin in 27 clinical centers around the United States for pre-diabetic subjects aged 25 years and above for a mean follow-up period of about 3 years [7]. Pre-diabetic subjects refer to subjects with impaired glucose tolerance (i.e. two hours glucose at 140–199 mg/dL after 75 g oral glucose tolerance test) and fasting plasma glucose between 95–125 mg/dL [7]. The DPP aimed to investigate whether onset of type 2 diabetes could be delayed or prevented via dietary changes and increased physical activity to achieve moderate weight loss or treatment with the oral metformin among study participants [8]. This study was modelled based on the DPP because it had investigated both lifestyle and pharmacological interventions as well as demonstrated that pre-diabetic subjects in the lifestyle modification group had a 58% reduction in risk of developing type 2 diabetes compared to placebo while that of those who took metformin were 31% less likely to develop type 2 diabetes compared to placebo [7].

In Singapore, significant resources have been spent on health promotion as part of a national health policy since a landmark report that strongly advocated healthy living in Singapore was published in 1991 [9]–[10]. As diabetes is an increasingly important public health issue, the cost-effectiveness of alternative strategies to prevent diabetes is of critical importance for planners and policymakers. Cost effectiveness analysis based on the DPP in the United States shows that neither lifestyle modification and metformin are cost-effective relative to placebo treatments over the immediate three-year horizon (although lifestyle modification may be cost-effective in the longer term) [11]. However, no such data is currently available for countries in Asia except in India [12]. Although the effects of the DPP were found to be similar in all racial and ethnic groups, due to substantial differences in health care systems and treatment costs across countries, economic results from US may not be generalizable to Asia; neither from India to Singapore [13]. Notably, in Singapore, medical costs and hence relative intervention costs are significantly lower than the United States while the socioeconomic standard and ethno-demographic composition of Singapore differs significantly from that of India [14]. Therefore, we sought to build upon previous studies to determine the anticipated 3-year cost per quality of adjusted life-years (QALYs) from 2010 to 2012 for metformin as well as lifestyle modification and compare it to no intervention in the Singapore healthcare setting.

## Methods

**Figure 1:**
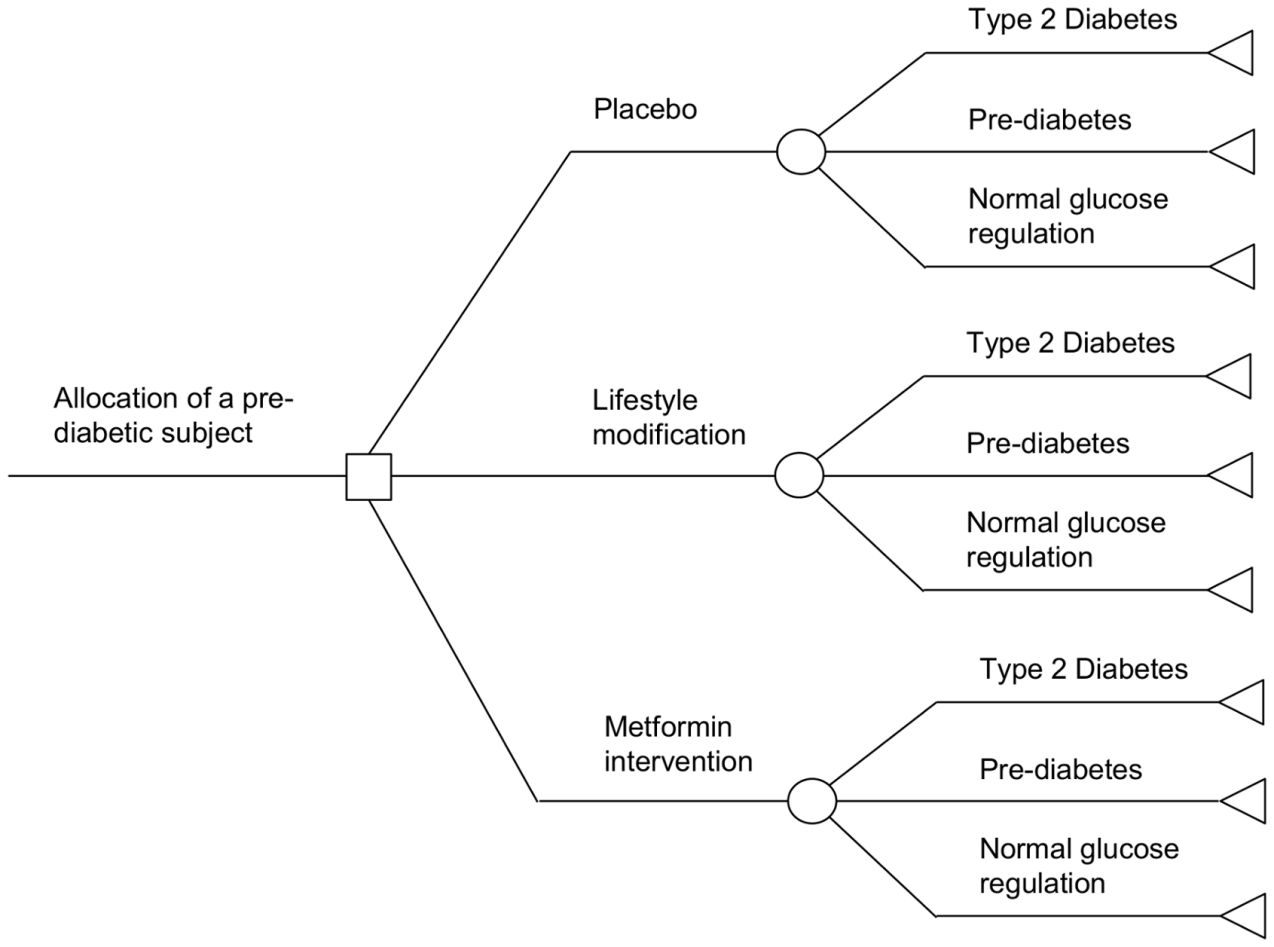
3-year decision tree for comparing cost-effectiveness of alternative interventions for a pre-diabetic subject.

A decision tree (Figure 1) was utilized to estimate the three-year costs and health outcomes. The decision tree was constructed with a decision node, three chance nodes and nine terminal nodes to represent the outcome. A three-year period was assumed as the average follow-up period of the DPP was three years and doing so would help to reduce unnecessary extrapolation of benefits beyond the period of observation [7]. We assumed that the intervention described in the DPP study or an intervention with similar effectiveness was replicated in Singapore. The model structure is based on the different health states that a pre-diabetic subject can progress into (i.e. normal glucose regulation, pre-diabetes and diabetes) [11]. Data from the DPP were used to estimate probability of health events, health-care costs, and health-related quality of life during a period of three years from 2010 to 2012 (Table 1). Linear extrapolation was used to estimate the probability of health events in normal glucose regulation state.

**Table 1:** Model input parameters for 3 years.

Parameter	Placebo	Lifestyle	Metformin	Source(s)
**(A) Probability of progressing to a health state**				
Type 2 diabetes	0.110	0.048	0.078	[7]
Pre-diabetes	0.826	0.804	0.831	Estimation
NGR*	0.064	0.148	0.091	[15]
**(B) Quality adjusted life years (QALYs)**				
Type 2 diabetes	1.97	1.91	2.02	[11]
Pre-diabetes/NGR	1.98	2.03	1.99	
**(C) Health system perspective** † **(2012US$)**				
Type 2 diabetes	14,275	15,555	14,780	[15]
Pre-diabetes/NGR	7,281	8,560	7,786	
**(D) Societal perspective** ‡ **(2012US$)**				
Type 2 diabetes	32,921	35,163	33,232	[16]–[22]
Pre-diabetes/NGR	25,867	28,108	26,177	

NGR: normal glucose regulation.

* Linear extrapolation used.

† Health system perspective = total direct medical cost = intervention+care outside DPP.

‡ Societal perspective = total direct medical cost+direct nonmedical cost+indirect cost.

### Cost estimations

Cost-effectiveness analyses were performed to compare different strategies to prevent diabetes (i.e. lifestyle modification and metformin) with no treatment, and included appropriate direct and indirect economic costs. For all the costs, we assumed Singapore pre-diabetic subjects to have similar utilization patterns and compliance rate as their US counterparts (i.e. similar items and units used in a period of time as well as participation rate). A 44-hour work week was assumed with 176 hours at work per month [23].

Direct medical costs consist of cost of medical services and goods from the perspective of health systems in order to implement and maintain the DPP interventions. Such costs involve cost of outpatient care, laboratory tests (i.e. fasting glucose, oral glucose tolerance test, venipuncture, serum and urine creatinine and hemoglobin/hematocrit) and medications (e.g. 850 mg of metformin). These costs were obtained from the National University Hospital cost repository based on the 9^th^ revision of the International Classification of Diseases (ICD9) codes corresponding to type 2 diabetes. The closest occupation matching the job description of the providers in US or the most likely substitute appropriate to the local setting was used when the same occupation title cannot be found in Singapore. For example, a medication case manager in the US was substituted by a registered nurse while a medical assistant was substituted by a healthcare assistant in Singapore.

Direct non-medical cost from the societal perspective was taken into account by estimating the participant time from the frequency and duration of encounters and calls reported by the DPP staff. In this study, we follow the assumptions from the DPP study that (i) participants spent an average of 30 minutes traveling to the DPP venue and back from the DPP appointments; (ii) travel time and time spent at DPP appointments will cost an equivalent of half of the median hourly wage of a person in 2010, and (iii) value of leisure time physical activity taken to be half of median hourly wage of a person when a participant disliked it, a quarter of a median hourly wage when a participant was neutral to it and at no cost if a participant liked it [24]. These time and travel assumptions are reasonable in the context of Singapore, a relatively small city-state.

As data showing individual utilization patterns across the three health statuses were not available in our study, we used the average monthly expenditure from the 2007/08 Household Expenditure Survey for the fitness equipment, household appliances to prepare food and food costs [16]. For expenditure under services, the maximum cost from a public facility was considered [17]–[19]. Furthermore, transportation cost was taken by multiplying the average number of hospital visits with the weighted-average transport costs to seek medical care [20]–[21].

However, direct medical cost outside DPP and indirect cost were not estimated using the methodology adopted by the DPP study. This is because we do not have the data to estimate the direct medical cost outside of the DPP by the three treatment groups and three health statuses. On the other hand, the DPP assumed that indirect cost incurred by the non-diabetic (i.e. subjects with normal glucose regulation level and pre-diabetic subjects) and diabetic patients were the same during the program while we believed that the indirect cost of diabetic patients would be different from the non-diabetic subjects. Hence a different methodology to differentiate the indirect cost between diabetic and non-diabetic subjects was applied.

Direct medical cost outside DPP was estimated using a ratio of 2 for diabetic to non-diabetic medical care expense as recommended by the IDF for industrialized countries [25]. Indirect cost was estimated from work days lost to diabetes (i.e. absenteeism). The average outpatient sick leave and hospitalization leave in the Singapore population were obtained from Ministry of Manpower [26]. In addition, a recent study of workplace safety and health commission by the Ministry of Manpower in Singapore showed that diabetic patients reported a mean difference of 1.3 days of sick leave higher than non-diabetics with no differences by age and gender [27]. Dropout rate and productivity losses from mortality were excluded as we assumed that both dropout rate and mortality rate from workforce will be relatively stable within 3 years. The indirect cost for non-diabetic subject was estimated by multiplying the median wage with the total number of days of sick leave and the proportion of the population who took sick leave [28]. Indirect cost for diabetic patient was estimated by adding the product of median wage with the mean difference of 1.3 days of sick leave to the indirect cost incurred by the non-diabetic subjects.

For direct medical and direct cost outside DPP, the initial costs were in 2010 SGD and were then inflated using the overall consumer price index in 2011 and 2012 while direct nonmedical cost and indirect cost were converted to net present value using a 3% discount rate [29]. Costs from health system perspective was calculated by summing the direct medical cost and direct medical cost outside DPP while costs from the societal perspective was calculated from summing the costs from health system perspective, direct nonmedical cost and the indirect cost. All costs here are standardized to 2012 Singapore dollars and expressed in 2012 United States dollars using a conversion rate of US$1:S$1.25 [30].

### Outcome assessment

Outcomes may depend on the care processes and the interventions under study hence QALYs from the US DPP was adopted [31]. The QALYs estimate was based on the Self-Administered Quality of Well-Being Index (QWB-SA) data collected within the US DPP study. QALYs combine quantity and quality of life by summing the product of the number of years of life and the quality of life, which is measured in health utilities, in each of those years. The QWB-SA assigns optimal functioning to be 1 and health deemed to be equivalent to death to be 0. Furthermore, the QWB-SA combines three scales (i.e. mobility, physical activity and social activity) with a measure of symptoms and functioning that may have affected the patient over the past three days from a list of 58 items to provide a health utility score [32]. In this study, we assumed normal glucose regulation to have the same QALYs as pre-diabetic subjects in this study and QALYs was discounted at a 3% rate before using it in the analysis. QALYs were discounted as the outcome occurred at the end of the study while cost was assumed to have incurred at the beginning of the study.

### Data analysis

Costs and QALYs were calculated over a three year period and are presented as mean outcomes per subject. The estimated mean costs and QALYs were combined into an incremental cost-effectiveness ratio (ICER) defined as: ICER = (C_1_–C_0_)/(Q_1_–Q_0_) where C is the estimated mean cost, Q the estimated mean QALYs, and the treatment strategies are indexed 1 for metformin intervention or lifestyle modification and 0 for placebo. ICERs were calculated using the adjusted costs and health utilities.

Several assumptions were made to simplify model construction and parameter estimation. In order to test the impact of these assumptions on the costs, deterministic sensitivity analysis (i.e. one-way analysis) was performed to assess the impact on the model’s conclusions by varying certain parameters. Several alternative scenarios were analysed to assess uncertainty in the cost-effectiveness results related to model assumptions and data inputs that were not associated with sampling uncertainty. Firstly, we varied the indirect cost by considering a low scenario of 0.5 days and a high scenario of 3 days as the American Diabetes Association (ADA) had reported an average excess absenteeism rate of 3 days for diabetics in 2012 [33]. Secondly, as we had assumed local diabetic patients to have similar utilization pattern and compliance rate as their US counterparts, a ratio of 2.3 for diabetic to non-diabetic medical care expense was adopted based on the figure from a study conducted by the ADA [33]. Lastly, the direct nonmedical cost was increased and decreased by 15% separately to assess the difference in relative costs.

For the QALYs, two separate one-way sensitivity analysis was conducted by individually varying the QALYs of metformin intervention and lifestyle modification by ±10%. A threshold analysis was then conducted in which the minimum incremental effectiveness compared to placebo that would meet a cost-effectiveness threshold of 1 GDP per capita, approximately US$53,000 in 2012 was computed. In addition, a two-way sensitivity analysis was done where the QALYs of metformin and lifestyle were simultaneously varied by ±10% in steps of 5% in order to determine the effectiveness between metformin intervention and lifestyle modification.

All analyses were performed using Microsoft Excel (Microsoft Corporation, Redmond, Washington, DC, US).

## Results

From the base-case analysis as depicted in Table 2, the direct medical cost was US$286, US$1,566 and US$791 for placebo, lifestyle modification and metformin intervention respectively. The relative magnitude of direct medical costs of the interventions compared to placebo using local pricing is hence significantly lower than in the US DPP study [24]. In addition, direct non-medical cost was US$18,140, US$19,102 and US$17,946 for placebo, lifestyle modification and metformin intervention respectively. Direct medical cost outside DPP was US$6,995 and US$13,989 for non-diabetic and diabetic patients respectively, regardless of the intervention. Likewise, indirect cost for non-diabetic and diabetic subjects amounted to US$446 and US$506 respectively. Thus, the total economic cost for non-diabetic subjects from the health system perspective was US$7,281, US$8,560 and US$7,786 for placebo, lifestyle modification and metformin intervention respectively. On the other hand, the total economic cost for diabetic patients from the health system perspective was US$14,275, US$15,555 and US$14,780 for placebo, lifestyle modification and metformin intervention respectively. The total economic cost for non-diabetic subjects from the societal perspective was US$25,867, US$28,108 and US$26,177 for placebo, lifestyle modification and metformin intervention respectively. For the diabetic patients, the total economic cost from the societal perspective was US$32,921, US$35,163 and US$33,232 for placebo, lifestyle modification and metformin intervention respectively.

**Table 2:** Breakdown of cost among individuals with and without diabetes from base-case analysis (in 2012US$).

	Without diabetes			With diabetes		
	Placebo	Lifestyle	Metformin	Placebo	Lifestyle	Metformin
Direct medical cost: intervention	286	1,566	791	286	1,566	791
Direct medical cost: care outside DPP	6,995	6,995	6,995	13,989	13,989	13,989
Direct nonmedical cost	18,140	19,102	17,946	18,140	19,102	17,946
Indirect medical cost	446	446	446	506	506	506
Cost from health system perspective	7,281	8,560	7,786	14,275	15,555	14,780
Cost from societal perspective	25,867	28,108	26,177	32,921	35,163	33,232

The base-case ICER of lifestyle modification compared to placebo was US$17,184 per QALY while that of metformin versus placebo was US$21,065 per QALY from the health system perspective (Table 3). On the other hand, the base-case ICER of the lifestyle modification relative to placebo was US$36,663 per QALY while that of metformin versus placebo was US$6,367 per QALY from the societal perspective (Table 4).

**Table 3:** Base-case cost-effectiveness analysis with deterministic sensitivity analysis from a health system perspective.

Intervention	QALY	Baseline scenario		High direct cost outside DPP scenario*	
		Cost	ICER	Cost	ICER
Placebo	1.98	8,050	–	8,281	–
Lifestyle	2.03	8,896	17,184	8,997	14,541
Metformin	1.99	8,331	21,065	8,495	16,030

QALY: quality adjusted life years; ICER: incremental cost-effectiveness ratio (in 2012US$ per QALY).

* Baseline+a ratio of 2.3 for direct cost of diabetic to non-diabetic medical care expense.

**Table 4:** Base-case cost-effectiveness analysis with deterministic sensitivity analysis from a societal perspective.

Intervention	QALY	Baseline scenario		Low productivity loss scenario*		High productivity loss scenario†		High direct cost outside DPP scenario‡		Low direct nonmedical cost scenario§		High direct nonmedical cost scenario||	
		Cost	ICER	Cost	ICER	Cost	ICER	Cost	ICER	Cost	ICER	Cost	ICER
Placebo	1.98	26,643	–	26,647	–	26,662	–	26,874	–	23,922	–	29,364	–
Lifestyle	2.03	28,447	36,663	28,449	36,609	28,455	36,440	8,548	34,019	25,581	33,730	31,312	39,596
Metformin	1.99	6,728	6,367	26,731	6,265	26,741	5,942	6,891	1,333	24,036	8,550	29,420	4,184

QALY: quality adjusted life years; ICER: incremental cost-effectiveness ratio (in 2012US$ per QALY).

* Baseline+workdays lost at 0.5 days.

† Baseline+workdays lost at 3 days.

‡ Baseline+a ratio of 2.3 for direct cost of diabetic to non-diabetic medical care expense.

§ Baseline+direct nonmedical cost decreased by 15%.

|| Baseline+direct nonmedical cost increased by 15%.

The one-way sensitivity analysis from the health system perspective showed that the ICER of lifestyle modification versus placebo to be lower than metformin versus placebo (Table 3). However, from the societal perspective, the ICER of metformin versus placebo was lower than that of lifestyle modification compared to placebo (Table 4). In addition, the ICERs from the societal perspective of lifestyle modification versus placebo were robust over a wide range of input parameters (with most changes to input parameters causing a change in ICER of no more than US$3,000 per QALY). Likewise, the ICERs from the societal perspective of metformin versus placebo were robust over a wide range of input parameters, causing a change in ICER of no more than US$2,200 per QALY except when the ratio of direct cost outside DPP of diabetic to non-diabetic subjects was raised to 2.3 and caused a change in ICER of US$5,035 per QALY.

Results from the one-way sensitivity analysis of the QALYs of metformin and lifestyle showed that since the extreme lower range of these values rendered both metformin and lifestyle clinically ineffective or having a negative incremental effect relative to placebo, any intervention at that range is dominated from a cost-perspective (Tables 5 and 6). The threshold analysis showed that a lifestyle modification will meet this criterion subject to a reduction of effectiveness of up to 0.02 QALYS (31% of the baseline assumed incremental effect), and a metformin intervention will meet this criterion subject to a reduction of 0.01 QALYS (or 88% of the baseline assumed incremental effect).

**Table 5:** One-way sensitivity analysis of lifestyle modification’s effectiveness.

	ICER of lifestyle modification (Health system)	ICER of lifestyle modification (Societal)
Cost of lifestyle modification	8,896	28,447
**QALY**		
1.82	Dominated	Dominated
1.93	Dominated	Dominated
2.03	17,184	36,663
2.13	5,617	11,984
2.23	3,357	7,162

QALY: quality adjusted life years; ICER: incremental cost-effectiveness ratio (in 2012US$ per QALY).

**Table 6:** One-way sensitivity analysis of metformin intervention’s effectiveness.

	ICER of metformin intervention (Health system)	ICER of metformin intervention (Societal)
Cost of metformin intervention	8,331	26,728
**QALY**		
1.79	Dominated	Dominated
1.89	Dominated	Dominated
1.99	21,065	6,367
2.09	2.489	752
2.19	1,323	400

QALY: quality adjusted life years; ICER: incremental cost-effectiveness ratio (in 2012US$ per QALY).

From the two-way sensitivity analysis of the QALYs, within the range of ±10% of QALYs of metformin and lifestyle modification, the choice between lifestyle and metformin varied. Given that lifestyle is more expensive, there is a range of effectiveness scenarios in which lifestyle would be less effective than metformin and hence strictly dominated. In the cases where lifestyle modification was at least as effective as metformin intervention, the relative ICER ranged between US$2,385 and US$17,491 from the health system perspective (Table 7) and between US$7,261 and US$53,253 from the societal perspective (Table 8).

**Table 7:** Two-way sensitivity analysis of the effectiveness between metformin intervention and lifestyle modification from health system perspective.

		QALY of lifestyle modification				
		1.82	1.93	2.03	2.13	2.23
**QALY of metformin intervention**	1.79	17,491	4,226	2,403	1,679	1,290
	1.89	Dominated	16,570	4,170	2,385	1,670
	1.99	Dominated	Dominated	15,742	4,115	2,367
	2.09	Dominated	Dominated	Dominated	14,992	4,062
	2.19	Dominated	Dominated	Dominated	Dominated	14,311

QALY: quality adjusted life years.

**Table 8:** Two-way sensitivity analysis of the effectiveness between metformin intervention and lifestyle modification from societal perspective.

		QALY of lifestyle modification				
		1.82	1.93	2.03	2.13	2.23
**QALY of metformin intervention**	1.79	53,253	12,866	7,317	5,112	3,928
	1.89	Dominated	50,450	12,696	7,261	5,085
	1.99	Dominated	Dominated	47,928	12,530	7,207
	2.09	Dominated	Dominated	Dominated	45,646	12,368
	2.19	Dominated	Dominated	Dominated	Dominated	43,571

QALY: quality adjusted life years.

## Discussion

Our study showed that both lifestyle modification and metformin intervention are worth implementing in Singapore as both are considered to be very cost-effective based on the World Health Organisation’s (WHO) benchmark where very cost-effective interventions was defined as having ICERs below 1×gross domestic product (GDP) per capita [34]. In addition, our study has also shown that both lifestyle and metformin interventions are likely to be associated with modest incremental costs compared with the placebo intervention [24]. These results are more favourable to intervention than the short-term findings from the US DPP, partly because in the local setting, the relative direct medical costs of both interventions are lower as compared to the placebo.

From the health system perspective, lifestyle modification had a lower ICER relative to placebo compared to metformin against placebo. However, from the societal perspective, metformin had a lower ICER than lifestyle modification when compared to placebo. In our analysis, this result is driven by the direct and indirect medical costs for lifestyle modification relative to metformin (US$1,566 in direct medical costs for lifestyle modification versus US$791 for metformin, and US$19,102 in indirect medical costs for lifestyle modification versus US$17,946 for metformin). These higher costs are mainly due to the cost of exercise equipment that was to be loaned to the subjects.

As health promotion is an important existing strategic priority for Singapore, we consider what goals a lifestyle modification program would need to set in order to become as cost-effective as a metformin intervention. We calculated that in order for this to happen, a lifestyle program would need to maintain the same level of effectiveness while reducing direct medical costs by at least 42% and direct nonmedical costs by 5%. This is calculated by varying the cost ratio of the direct medical cost and that of the direct nonmedical cost of lifestyle modification. Thus, very significant cost-saving innovations would need to be realized in order for lifestyle modification to dominate metformin from a societal perspective, an important consideration for decision makers.

This study is the first to estimate the cost of metformin and lifestyle modification if a program similar to DPP is implemented in Singapore. However, this study is subjected to the following limitations. Firstly, as Singapore’s GDP per capita is high at US$65,048 while the proportion of GDP spent on healthcare is low (i.e. 4% GDP), the WHO benchmark might not be applicable [35]–[36]. A better approach might be to plot a cost-effectiveness acceptability curve (CEAC) to deal with the uncertainty in the estimates of cost-effectiveness. However, it cannot be used to make statements about the implementation of the intervention, which is our main objective; hence a CEAC was not plotted in this study [37]. Secondly, the cost assumptions used in the model may not hold true but since the results from the sensitivity analysis are robust, the assumptions can be taken to be adequate. Thirdly, since the model is based on the overall DPP study results, the ICER may be affected if clinical events and outcomes of pre-diabetic subjects in Singapore differ significantly from it. The sensitivity analyses conducted on the QALYs showed that ICER is relatively sensitive to estimates of effectiveness even though the lifestyle and metformin interventions are both likely to be cost-effective under scenarios that are plausible for Singapore given our reading of the current literature. Thus, we suggest that future research should focus on establishing effectiveness in the local setting. Fourthly, the scope of our study is focused on diabetes prevention only and does not perform other comparisons between interventions. Although lifestyle modification has a higher ICER relative to placebo than that of metformin intervention in this analysis, it should be noted that since individuals with pre-diabetes are also at an increased risk for developing cardiovascular diseases, lifestyle modification could potentially also reduce the risk of such diseases better than a metformin intervention as demonstrated in another study conducted by the DPP group [38]. Lastly, our results may not be generalizable to low- and middle-income countries (LMICs) due to different clinical management or LMICs having inadequate resources in diabetes care as well as health economic evaluation being context-specific.

In conclusion, based on the analysis in our study, both lifestyle modification and metformin intervention are likely to be cost-effective and worth implementing in Singapore even over a short horizon of three years. However, the cost of lifestyle modification from the societal perspective would have to be reduced in order to match the cost-effectiveness of metformin.
