# Correction: Evaluating the Cost-Effectiveness of Lifestyle Modification versus Metformin Therapy for the Prevention of Diabetes in Singapore

**DOI:** 10.1371/journal.pone.0120831

**Published:** 2015-03-16

**Authors:** The PLOS ONE Staff

In Table 6, the value of ICER of metformin intervention (Health system) at QALY = 2.09 should be 2,489. Please see the corrected Table 6 here.

**Table 6:** One-way sensitivity analysis of metformin intervention’s effectiveness

	ICER of metformin intervention (Health system)	ICER of metformin intervention (Societal)
**Cost of metformin intervention**	8,331	26,728
**QALY**		
1.79	Dominated	Dominated
1.89	Dominated	Dominated
1.99	21,065	6,367
2.09	2,489	752
2.19	1,323	400

QALY: quality adjusted life years; ICER: incremental cost-effectiveness ratio (in 2012US$ per QALY)

## References

[1] Png ME, Yoong JS-Y (2014) Evaluating the Cost-Effectiveness of Lifestyle Modification versus Metformin Therapy for the Prevention of Diabetes in Singapore. PLoS ONE 9(9): e107225 doi: 10.1371/journal.pone.0107225 25203633 10.1371/journal.pone.0107225 PMC4159303

